# Characterization of Five CRISPR Systems in *Microcystis aeruginosa* FACHB-524 with Focus on the In Vitro Antiviral Activity of One CRISPR System

**DOI:** 10.3390/ijms26041554

**Published:** 2025-02-12

**Authors:** Mengjing Zeng, Qi-Ya Zhang, Fei Ke

**Affiliations:** 1 Institute of Hydrobiology, Chinese Academy of Sciences, Wuhan 430072, China; zengmengjing@ihb.ac.cn(M.Z.);; 2 College of Advanced Agricultural Sciences, University of Chinese Academy of Sciences, Beijing 100049, China

**Keywords:** *Microcystis aeruginosa*, CRISPR-Cas system, type III-B CRISPR system, Cmr-crRNA effector complex, in vitro expression, anti-phage activity

## Abstract

*Microcystis aeruginosa* is an important species causing cyanobacterial blooms, which can be effectively infected and lysed by cyanophages. Several strategies have been developed by *M. aeruginosa* to resist cyanophage infections, including the CRISPR-Cas systems. However, detailed information on the CRISPR-Cas systems in *M. aeruginosa* is rare. In the present study, the CRISPR-Cas systems of *M. aeruginosa* FACHB-524 were analyzed by genome re-sequencing, which showed that there are two type I (Cluster 1, I-B1; Cluster 2, I-D) and three type III-B (Cluster 3/4/5) CRISPR-Cas systems in the cyanobacteria. Further comparison revealed that spacer sequences of two type III-B systems targeted several genes of the cyanophage MaMV (*M. aeruginosa* myovirus) strains. One of the type III systems (Cluster 4) was then cloned and expressed in *Escherichia coli* BL21 (DE3). Protein purification and mass spectrometry identification revealed that a Cmr-crRNA effector complex formed in the *E. coli*. Subsequently, *T4 phage (T4)* was used to infect the *E. coli,* expressing the Cmr-crRNA complex with or without accessory proteins. The results showed that the Cmr-crRNA effector complex exhibited anti-phage activity and the accessory protein Csx1 enhanced the immune activity of the complex. Collectively, our results comprehensively demonstrate the CRISPR systems encoded by a strain of *M. aeruginosa*, and for the first time, one of the CRISPR systems was constructed into *E. coli*, providing a foundation for further in-depth analysis of cyanobacterial CRISPR systems.

## 1. Introduction

*Microcystis* is a genus of freshwater cyanobacteria, which includes species that can cause harmful algal blooms in freshwater ecosystems worldwide, such as *Microcystis aeruginosa* (*M. aeruginosa*) [1,2,3]. Several strains of *M. aeruginosa* have the ability to produce toxic secondary metabolites, such as microcystin, which is a potent hepatotoxin [4]. Thus, algal blooms have caused long-term health hazards to humans by jeopardizing the safety of drinking water, posing multiple threats to economic and social development [5]. Cyanophages can specifically infect and lyse cyanobacteria [6], making the use of cyanophages for the control of cyanobacteria an attractive and promising idea [7,8]. Currently, multiple cyanophages have been isolated from *Microcystis* [9], such as MaMV-DH01 [10], MaMV-DC [11] and Mic1 [12].

CRISPR-Cas (clustered regularly interspaced short palindromic repeats (CRISPR)–CRISPR-associated (Cas)) systems are widely distributed among archaea and bacteria, serving as an adaptive immune system that protects these microorganisms from phage and plasmid attacks. The working principle of CRISPR-Cas system involves integrating short sequences from the target genome (spacers) into its own CRISPR locus [13]. It typically uses short CRISPR RNAs (crRNAs) derived from repetitive/spacer sequences. These small crRNAs, which carry most or all of the spacer sequences, act as guide RNAs for various interference modules that target and cleave DNA or RNA with the help of Cas proteins, thereby silencing foreign nucleic acids in a sequence-specific manner [14,15]. Based on the classification of the Cas gene and the characterization of the crRNA-Cas effector complexes, CRISPR-Cas systems have been divided into two classes, which further can be divided into six types and thirty-three subtypes [16]. Class 1 systems feature multi-subunit crRNA-effector complexes, including types I, III, and IV, while in Class 2 systems, all functions of the effector complex are carried out by a single protein (such as Cas9), including types II and V. Type I CRISPR-Cas systems are currently classified into seven subtypes: I-A, I-B, I-C, I-D, I-E, I-F, and I-U. The type III CRISPR-Cas systems are classified into six subtypes, from III-A to III-F [16].

The immune mechanisms of the bacterial CRISPR system exhibit diversity in both structure and function, with this diversity primarily observed between type I and type III systems [17]. The structure and function of type III systems are more diverse than those of type I systems. While type I systems target DNA, type III-A and III-B systems are associated with DNA and RNA interference, respectively [18,19]. However, the RNA-targeting nature of type III-B system is not absolute. For instance, studies in *Sulfolobus islandicus* suggest that the interference mediated by the III-B complex Cmr-α depends on antisense transcription derived from the original spacer region, but it appears to target DNA. In addition, the Cas6 gene encodes an enzyme that specifically recognizes CRISPR-derived transcripts and processes them into functional crRNAs, which serve as guides for interference complexes [20,21].

Analysis of cyanobacteria genomes revealed that the CRISPR-Cas systems were widely distributed among cyanobacteria, including type I-A, I-B, I-C, I-D, I-E, III-A, and III-B, reflecting that phages are an important force driving the evolution of cyanobacterial genomes [22,23]. Analysis of Cas1 protein revealed that I-D and III-B subtype systems may be conserved in freshwater unicellular cyanobacteria. However, no type II CRISPR-Cas system was found in cyanobacteria genomes until now. For the CRISPR-Cas system of *Microcystis*, the Cas gene sets of I-A, I-D, III-A, and III-B have been found [24,25]. After being infected with the cyanosiphophage Mic1, it was found that most genes of type III-B CRISPR system in *M. aeruginosa* FACHB-1339 were significantly upregulated at the transcriptional level, indicating that the invasion of Mic1 activated host’s CRISPR-Cas defense system [26].

Previously, we have identified CRISPR elements targeting the cyanophage MaMV-DC (*M. aeruginosa* myovirus from Lake Dianchi) in the genome of *M. aeruginosa* FACHB-524 [11]. These CRISPR elements belong to type I-B, type I-D, and type III-B. However, the obtained CRISPR system was not entirely possible due to sequencing reasons. For example, the type I-B CRISPR system lacks the Cas4 protein subunit. In the present study, to gain a more comprehensive and in-depth understanding of the CRISPR system in *M. aeruginosa*, we re-sequenced the genome of *M. aeruginosa* FACHB-524 and analyzed its CRISPR systems. Furthermore, a type-III CRISPR-Cas system that may target MaMVs was successfully reconstructed and expressed in *Escherichia coli* (*E. coli*). Further investigation of the defense role of the type III-B CRISPR system against *Enterobacteria phage T4 (T4)* infection provided a valuable foundation for further exploration of the antiviral mechanisms of the *M. aeruginosa* type III-B CRISPR system.

## 2. Results

### 2.1. M. aeruginosa FACHB-524 Genome Sequence Analysis

**Figure 1:**
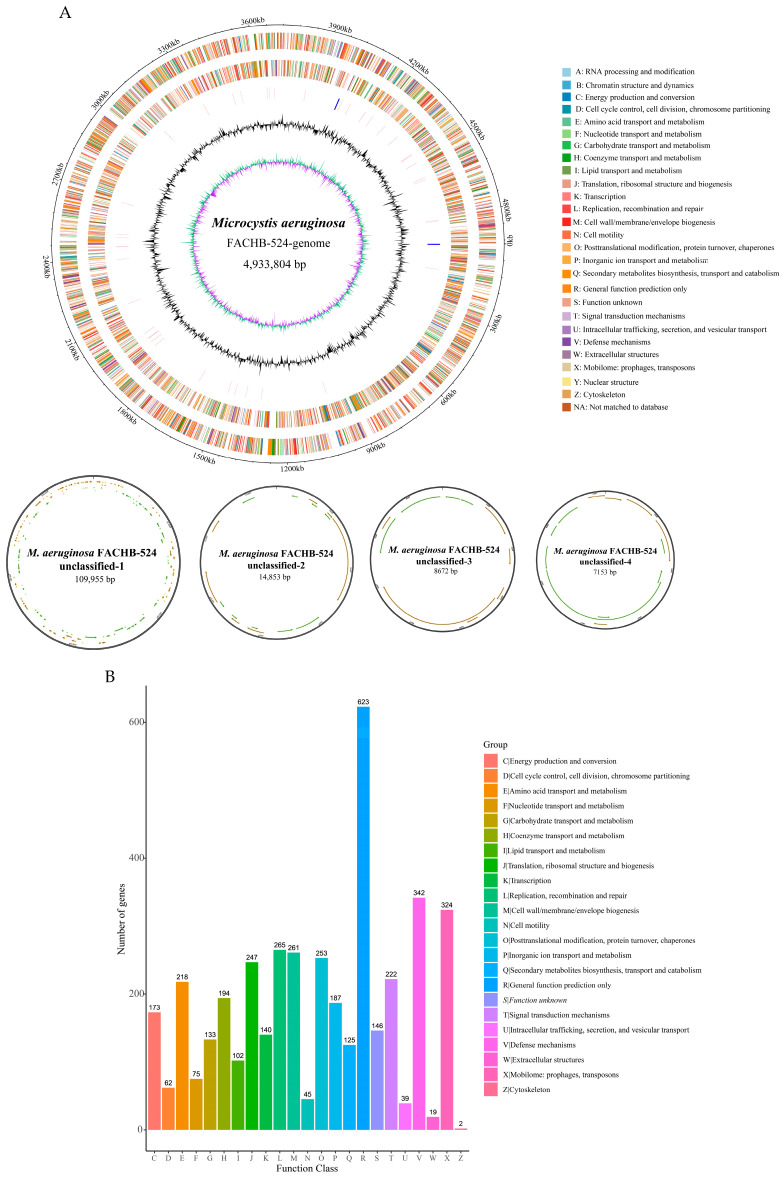
Genome circle diagram of *M. aeruginosa* FACHB-524 and gene function classifications. (**A**) Genome circle diagram. The circular diagrams from outside to inside indicate: 1. genome coordinates; 2. genes on the positive strand, with different colors representing different COG functional classifications; 3. genes on the negative strand; 4. rRNAs and tRNAs, with rRNAs being blue and tRNAs being red; 5. GC content curve, with 2000 bp as a sliding window; 6. GC skew curve, with a sliding window of 2000 bp; green indicates that the G content is greater than the C content, and violet indicates that the G content is less than the C content. (**B**) Gene function classification by COG analysis.

The current genomic sequencing results of *M*. *aeruginosa* FACHB-524 are not yet complete. To obtain the complete genome sequence of *M. aeruginosa* FACHB-524, we used nanopore sequencing and Illumina sequencing simultaneously. After filtering adapters, short fragments, and low-quality data, nanopore sequencing yielded 103,183 reads for a total of 982,441,195 bp of clean data for assembly, and 8,549,322 clean reads for a total of 1,276,206,792 bp of clean data were obtained by Illumina sequencing. The sequencing depth was ≥100×, indicating that the assembly result had good integrity. The complete genome sequence of *M*. *aeruginosa* FACHB-524 was determined from the assembly results (Table 1), which consists of a circular chromosome of 4,933,804 bp and four circular plasmids, with sizes of 109,955 bp, 14,853 bp, 8672 bp, and 7153 bp, respectively (Figure 1). The circular chromosome is named *M. aeruginosa* FACHB-524-genome, and the four plasmids are designated *M. aeruginosa* FACHB-524 unclassified-1/2/3/4. The complete genome sequence contains a total of 5071 genes. The *M. aeruginosa* FACHB-524-genome includes 4828 protein-coding genes, 4 rRNA operons, and 41 tRNA genes, with a GC content of 42.49%. The plasmids *M. aeruginosa* FACHB-524 unclassified-1/2/3/4 contain 138, 19, 10, and 8 protein-coding genes, with GC contents of 42.96%, 40.85%, 43.20%, and 44.65%, respectively (Table 1).

To annotate and classify the functions of genes in the *M. aeruginosa* FACHB-524 genome, we performed a Cluster of Orthologous Groups (COG) of proteins analysis. COG results showed that 4197 genes were classified into the COG family (82.76% of the *M. aeruginosa* FACHB-524 complete genome), which were further divided into 23 groups, including metabolism (e.g., E, F, and G), cellular processes (e.g., D, N, and U), function unknown (S), and so on (Figure 1B). Among these, a total of 146 genes were annotated under the unknown function category. Additionally, 342 genes were categorized under the defense mechanism group (V), including the *mrr* gene and *hsdS_1* gene, both of which are capable of functioning within the restriction–modification (RM) system, another important bacterial defense mechanism besides the CRISPR system.

### 2.2. CRISPR Systems Encoded by M. aeruginosa FACHB-524

**Figure 2:**
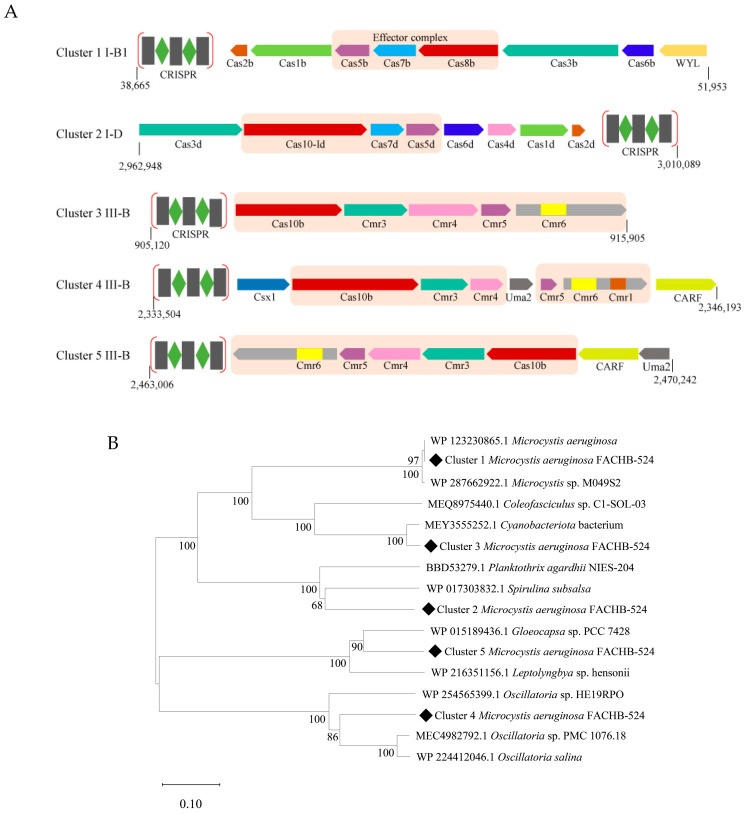
The CRISPR-Cas systems in the genome of *M. aeruginosa* FACHB-524. (**A**) Schematic diagram of the CRISPR-Cas systems. The Cas genes or accessory genes were indicated by arrows with homologous genes highlighted in the same color and that constitutes Cmr-α effector complex was labeled by light orange color box. The numbers indicate the CRISPR-Cas loci in the genome. (**B**) Phylogenetic analysis of Cas10 homologues in *M. aeruginosa* FACHB-524. The phylogenetic tree was constructed by neighbor-joining method with 1000 bootstraps.

We analyzed the CRISPR systems based on the assembled *M. aeruginosa* FACHB-524 genomes. The results indicate that *M. aeruginosa* FACHB-524 contains five CRISPR-Cas loci, including two type I systems (I-B1 and I-D) and three type III systems (III-B). These loci are named according to their position in the genome as Cluster 1 (I-B1), Cluster 2 (I-D), Cluster 3 (III-B), Cluster 4 (III-B), and Cluster 5 (III-B) (Figure 2A). Notably, Cluster 1 is located at 38,665–51,953 bp in the contig *M. aeruginosa* FACHB-524 unclassified-1. Clusters 2–5 are located in *M. aeruginosa* FACHB-524-genome. The positions of Clusters 2–5 in the genome are 2,962,948–3,010,089 bp, 905,120–915,905 bp, 2,333,504–2,346,193 bp, and 2,463,006–2,470,242 bp, respectively. Table 2 lists the CRISPR arrays and repeat sequences predicted by CRISPRLeader for Clusters 1–5, which shows that the number of spacer sequences between these five CRISPR-Cas loci ranges from 4 to 200.

As shown in Figure 2A, Cluster 1 is a type I-B1 CRISPR-Cas system, consisting of eight CRISPR-Cas protein subunits: Cas2b, Cas1b, Cas5b, Cas7b, Cas8b, Cas3b, Cas6b, one of which contains a WYL domain. The core subunit is composed of three proteins: Cas5b, Cas7b, and Cas8b. Cluster 2 belonged to the type I-D systems, which also contains eight CRISPR-Cas proteins, including Cas3d, Cas10-Id, Cas7d, Cas5d, Cas6d, Cas4d, Cas1d, and Cas2d, and the core subunits are Cas5d, Cas7d, and Cas10-Id.

Clusters 3–5 all belonged to type III-B CRISPR system. As a general rule, proteins of the type III-B CRISPR system contain homologues of Cmr1, Cas10 (also known as Cmr2), Cmr3, Cmr4, Cmr5, and Cmr6 which constitute the core effector complex, and other accessory proteins [27]. Interestingly, the core subunit Cmr1 of the Cmr-α effector complex was not predicted by using the PADLOC online tool. We then used NCBI’s CDD to predict the conserved domains of these proteins. The results showed that the Cmr1 and Cmr6 domains were located in a single protein in Cluster 4 (Figure 2A), and thus the protein was named as Cmr1-6. In contrast, only the conserved domain of Cmr6 was identified in both Cluster 3 and Cluster 5. Therefore, the core protein subunits of Cluster 3 include homologues of Cas10, Cmr3, Cmr4, Cmr5, and Cmr6. Cluster 4, in addition to the core subunits, includes three accessory protein subunits: homologues of Csx1, Uma2, and a protein containing CARF domain. CARF refers to a protein containing the CARF (CRISPR-Cas Associated Rossmann Fold) domain. Compared with Cluster 3, Cluster 5 has a homolog of Uma2 and a protein containing the CARF domain.

The homologues of Cas10 and Cas8 in Clusters 1–5 occupy similar positions in their respective complexes. It has been shown that the HD domains of both Cas8 and Cas10 exhibit single-stranded DNA nuclease activity [16,28]. Then, a phylogenetic tree for Cas8 and Cas10 from Clusters 1 to 5 was constructed to study their evolutionary relationship (Figure 2B). The phylogenetic analysis revealed that the Cas8 and Cas10 homologues of Clusters 1–5 exhibit distinct evolutionary relationships, as they distributed in different clades. Only the Cas8 homolog of Cluster 1 clustered closely with that from other strains of *M. aeruginosa.* Moreover, the amino acid sequences of Cas10/Cmr3/Cmr4/Cmr5/Cmr1-6 homologues from Clusters 3, 4, and 5 were aligned using BLASTP. Sequence identities among the Cas10 homologues ranged from 27.04% to 40.71%, with the highest identity was observed between Clusters 3 and 5. The sequence identity of the four proteins (Cmr3, Cmr4, Cmr5, and Cmr1-6) are provided in the Supplementary Information Table S1.

### 2.3. M. aeruginosa FACHB-524 Contains CRISPR Systems Targeting MaMVs

**Figure 3:**
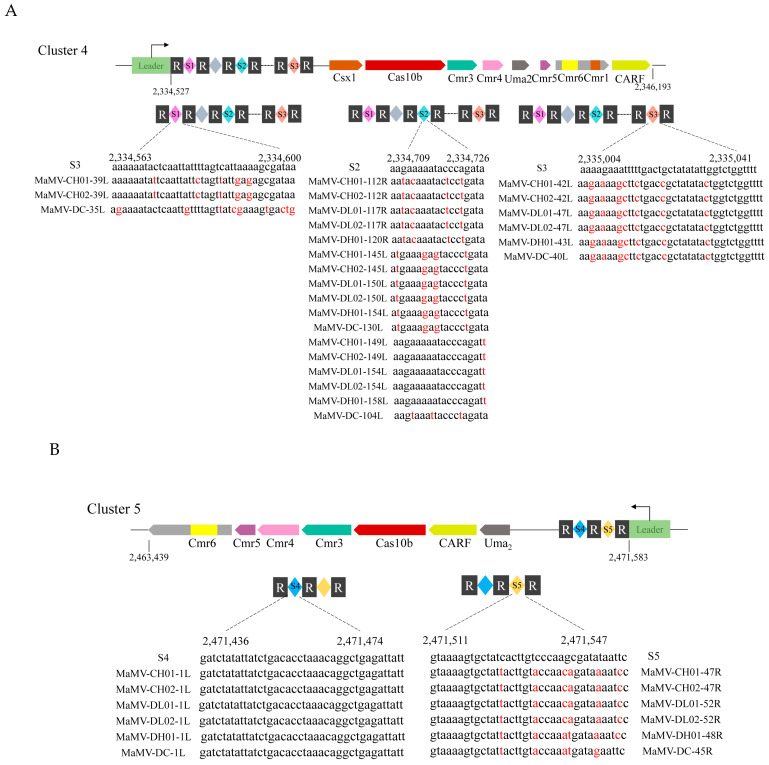
Targeting of *M. aeruginosa* FACHB-524 CRISPR system with genome sequences of MaMVs. (**A**) Sequences in Cluster 4 that target the MaMV genes. (**B**) Sequences in Cluster 5 that target the MaMV genes. The red highlighted bases indicate the bases in the spacer sequences differ from the genomes of the six MaMVs.

The diverse CRISPR-Cas systems and a large number of CRISPR spacer sequences in the *M. aeruginosa* FACHB-524 genome indicate that it could be frequently infected by various cyanophages. Our previous studies have isolated several MaMV strains (MaMV-CH01, MaMV-CH02, MaMV-DL01, MaMV-DL02, MaMV-DH01, and MaMV-DC) that infect *M. aeruginosa* FACHB-524 [10,11]. By comparing the CRISPR sequences with the genomes of these six MaMVs, it was found that some of the CRISPR spacer sequences in Cluster 4 and Cluster 5 are highly similar or even identical to certain protein encoding sequences of the six MaMVs (Figure 3). There are three spacer sequences of Cluster 4 that are highly similar to the genomes of the six MaMVs. For example, the S1 sequence matches MaMV-CH01-*39L*, MaMV-CH02-*39L*, and MaMV-DC-*35L* (Figure 3A). For the CRISPR-Cas system in Cluster 5, there are two spacer sequences that are highly similar to the genomes of the six MaMVs. For example, the S5 sequence (2,471,511–2,471,547 nucleotides) matches MaMV-CH01-*47R*, MaMV-CH02-*47R*, MaMV-DL01-*52R*, MaMV-DL02-*52R*, MaMV-DH01-*48R*, and MaMV-DC-*45R* (Figure 3B). The S4 spacer sequence is completely identical to the DNA sequence of the *1L* gene of the six cyanophages, which was predicted to encode a rIIA-like protein.

### 2.4. The Cluster 4 CRISPR-Cas System Was Successfully Expressed in E. coli

**Figure 4:**
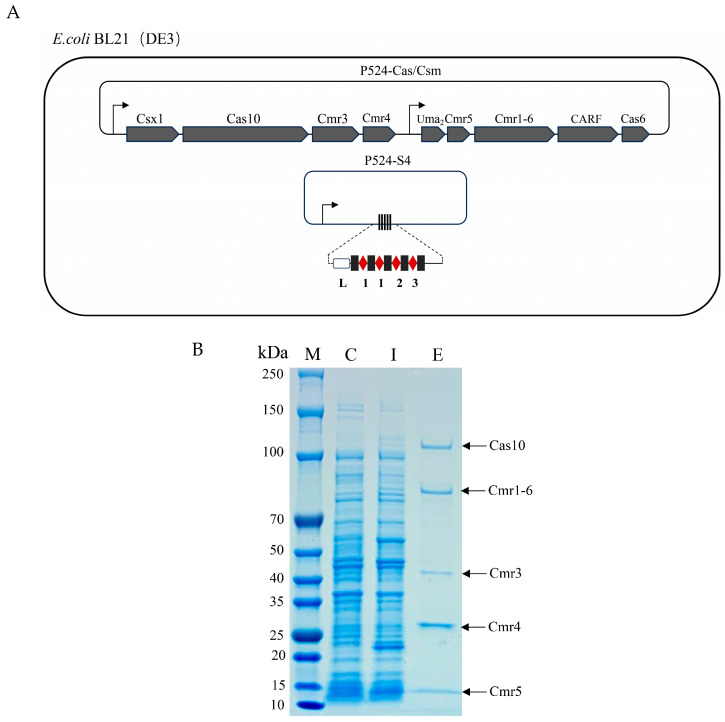
Expression and purification of the MaCmr complex in *E. coli*. (**A**) The strategies for construction recombinant plasmids. Four spacers were constructed into the P524-S4 plasmid. Arrows indicate the T7 promoter, and 1, 2, and 3 indicate different spacer sequences. (**B**) SDS-PAGE analysis of the purified protein complex. The composition of the Cmr-α effector complex was Cas10, Cmr1-6, Cmr3, Cmr4, and Cmr5 in descending order. C, lysates before IPTG induction; I, lysates after IPTG induction; E, the purified Cmr-α protein complex.

Because the genetic transformation system of *M. aeruginosa* was not yet established, we chose to construct the CRISPR system into *E. coli* BL21 (DE3) for prokaryotic expression. The Cluster 4 CRISPR system was selected as it possesses a longer Cas10 homolog than that of Cluster 5 and a Cmr1 domain. Based on the composition of the Cmr-α effector complex, we constructed two plasmids. One contains the nine genes encoding the core protein subunit of the Cmr-α effector complex (Cas10, Cmr3, Cmr4, Cmr5, and Cmr1-6) and Csx1, Uma2, CARF, and Cas6, the other contains the CRISPR array that can be transcribed and processed into mature crRNAs (Figure 4A). Theoretically, the crRNAs could be combined with the five core protein subunits to form a Cmr-α effector (MaCmr) complex in *E. coli* BL21 (DE3) cells. SDS-PAGE results showed that there were four distinct bands in addition to the 10His-tagged Cmr1-6 protein subunit after series purification, which were expected to be Cas10, Cmr3, Cmr4, and Cmr5 of the Cmr-α effector complex based on size and molar ratio (Figure 4B). Further, each protein band was subjected to mass spectrometry identification, and the results were consistent with expectations, confirming that they were the effector proteins expressed in prokaryotic systems (Table S2).

### 2.5. The MaCmr Complex Is Able to Form a Defense Barrier with Anti-Phage Activity

**Figure 5:**
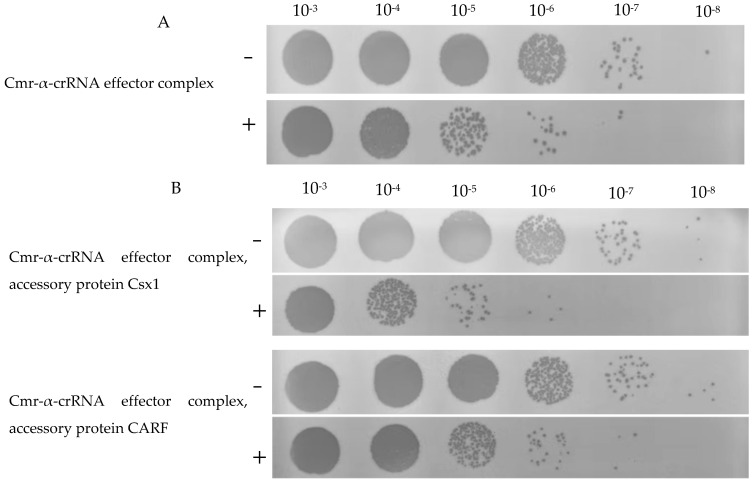
The titer of T4 phage in E. coli carrying different prokaryotic expression vectors. (**A**) Titers of T4 phage in E. coli expressing the Cmr-α-crRNA effector complex. (**B**) Titers of T4 phage in E. coli expression of both the Cmr-α-crRNA effector complex and the accessory protein Csx1 or CARF. “+” and “−” indicates E. coli was induced with or without IPTG.

By co-culturing the *T4 phage* with *E. coli* containing different transformants, the viral defense activity of the *M. aeruginosa* CRISPR system was investigated. The transformants contain different plasmid combinations expressing the Cmr-α effector complex proteins (Cas10/Cmr3/Cmr4/Cmr5/Cmr1-6), crRNA, or accessory proteins. The phage assay results showed that different transformed strains of *E. coli* exhibited varying degrees of antiviral defense following infection with *T4 phage*. As shown in Figure 5A, *E. coli* containing the Cmr-α effector complex with crRNA demonstrated a 10-fold increase in resistance to *T4 phage* infection compared to the control. Figure 5B indicates that the addition of the accessory protein Csx1 enhanced the *T4 phage* resistance activity by about 10-fold compared to Figure 5A, whereas the inclusion of the accessory protein CARF did not significantly enhance the defense capabilities of the CRISPR system. These results suggest that in the type III-B CRISPR system of *M. aeruginosa* FACHB-524 (Cluster 4), the presence of the Cmr-α effector complex allows the system to provide some defense against foreign viruses, and that Csx1 may enhance immunological activity.

## 3. Discussion

*Microcystis*, as the main cyanobacteria that causes algae blooms in freshwater systems, has received widespread attention. However, interactions between *Microcystis* and their cyanophages are largely unknown. In the present study, the CRISPR defense systems of *M. aeruginosa* FACHB-524 was investigated based on a re-sequencing of its genome. The results revealed that only type I and type III CRISPR systems were found in its genome, including one type I-B, one type I-D, and three type III-B, which was in accordance with previous reports stating that cyanobacteria only contain type I and III CRISPR systems [23]. For the five CRISPR systems, there are multiple spacers. For example, there are 200 spacers for the CRISPR system of Cluster 2. Spacers provide a genetic memory bank of previous invaders [16,29]. The multiple spacers showed the diversity of exogenous plasmids or cyanophages invading *M. aeruginosa* FACHB-524, which reflected the dynamic interactions and co-evolution between *M. aeruginosa* and cyanophages.

Several Cas proteins were revealed in the present analysis. It has been reported that the operons of type I and type III CRISPR systems are capable of encoding multiple Cas proteins [30]. Type I-B effector complexes are composed of Cas5, Cas7, and Cas8, while type I-D effector complexes consist of Cas5, Cas7, and Cas10. In contrast, type III-B effector complexes include Cas10, Cmr3, Cmr4, Cmr5, and Cmr6 or a fusion of Cmr1 and Cmr6. Although the domain of Cmr1 was not found in Cluster 3 and 5 of *M. aeruginosa* FACHB-524, whether the two Cmr6 homologues in Cluster 3 and 5 executed a function of Cmr1 needs further research, considering the length of the two homologues is similar with the Cmr1-6 of Cluster 4. Compared to the I-B system, the I-D system contains a Cas10d protein. As our research progressed, the I-D subtype was identified as a potential evolutionary intermediate between type III and type I systems [16]. The large subunits Cas8 and Cas10 in the I-B1 (Cluster 1) and I-D (Cluster 2) effector complexes occupy similar positions within the complex, their sequences are not homologous, and they perform distinct roles in target interference. The divergence of the large subunits Cas10 and Cas8 suggests that they may have evolved under stronger evolutionary pressures, particularly from phage anti-defense strategies [31].

Our sequence and phylogenetic tree analyses also illustrate the diversity of the CRISPR system. Although there are three Type III-B systems in the *M. aeruginosa* FACHB-524 strain, their core protein Cas10 is located on different branches of the phylogenetic tree. The sequence amino acid identities of their effector proteins are also not high. These findings suggest that the three type III-B systems may have different origins or have been subjected to different selective pressures during evolution.

There are several accessory proteins in the CRISPR systems of the present study. Compared to the core Cas genes, some accessory genes are more loosely associated with the CRISPR-Cas system. Accessory genes are more common and diverse in type-III CRISPR-Cas system. For instance, in Clusters 4 and 5, three accessory genes—*Uma2*, *Csx1*, and *CARF*—are present. The most common gene in accessory genes is the Csx1-like gene, which encodes the protein Csx1 [16]. Csx1 plays an accessory role during interference by non-specifically degrading exogenous transcripts, even when they are not part of the effector complex. The cOA-mediated activation of the accessory protein Csx1 is crucial for the immune response in type III CRISPR-Cas systems [32,33,34,35]. The sequence located in the protospacer region of this study is part of the viral late gene. When the target gene is transcribed during the late stage of the viral lifecycle, the activation of CRISPR-Cas type III-B related cOA synthesis and the subsequent activation of the non-specific ribonuclease Csx1 are required. We also identified an accessory protein with a WYL domain in the type I-B1 CRISPR-Cas system. Proteins containing the WYL domain are not uncommon in bacteria and have been reported to regulate the transcription of CRISPR-Cas systems. Furthermore, they are involved in microbial defense [36]. Further investigation is needed to explore the impact and mechanisms of the accessory proteins like Csx1, WYL, and CARF in *M. aeruginosa*.

Genetic manipulation, such as gene deletion or addition, is not available in *Microcystis* currently, so the model organism *E. coli* was used to express and evaluate one of the CRISPR systems. Reconstruction of CRISPR systems in *E. coli* has been reported for other bacteria or archaea [37,38], and discussed in the present study. Following the *T4 phage* infection showed that the Cmr-α-crRNA complex inhibited the multiplication of phage. It has been reported that type III-B CRISPR systems not only mediate transcription-dependent DNA interference but also RNA interference, both exhibiting antiviral defense activity [39,40]. In *Saccharolobus islandicus*, it was found that the complementarity between the crRNA and the target RNA in the purified natural Cmr-α complex is essential for the RNase and single-stranded DNase (ssDNA) activities of Cmr-α [40,41]. The Cmr-α-crRNA effector complex is endowed with the ability to eliminate foreign nucleic acids, thereby providing immunity against external invaders. In this study, we hypothesize that the Cmr-α-crRNA complex cleaves either the *T4 phage* genome or its transcribed RNA, thereby conferring immunity against *T4 phage* invasion. In the absence of Csx1, the Cmr-α-crRNA effector complex alone was sufficient to defend against phage infection. It is hypothesized that the type III-B CRISPR-Cas system effector complex, including the core subunit Cmr4, uses aspartic acid residues to catalyze the cleavage of bound target RNA at fixed six-nucleotide spacers [41], although further studies are needed to investigate the cleavage sites and catalytic activity of Cmr4.

In addition, our experiments also showed that the addition of the accessory protein Csx1 enhanced the immunity of *E. coli* against phage. After binding to the target RNA, Cmr-α complex was reported to activate the catalytic Cas10 subunit and generate the second messenger cOA [42]. The cOA, in turn, activates the CARF domain of Csx1 [43], enabling Csx1 to exert nonspecific nucleolytic activity, degrading RNA indiscriminately. Csx1 may also cause the nonspecific degradation of key biomacromolecules, potentially inducing cell dormancy or progression [44,45], thereby enhancing the antiviral defense response. However, the exact antiviral mechanism of Csx1 in this study requires further experimental investigation. In addition, although our results indicated that the CRISPR complex has an inhibitory effect on viral infection, the work was conducted in *E. coli*. Further functional validation and analysis are needed in algae in the future.

## 4. Materials and Methods

### 4.1. Algae and Phage Strains

*M. aeruginosa* FACHB-524 was obtained from the Freshwater Algae Culture Collection at the Institute of Hydrobiology (FACHB, Wuhan, Hubei, China), which is cultured in sterile BG11 medium as described previously [45]. Briefly, the cyanobacteria were cultivated in a constant temperature incubator at 25 °C with a photoperiod of 14 h light/10 h dark cycle using light intensity of approximately 30 μE·m^−2^·s^−1^, and gently shaken three times a day.

*Enterobacteria phage T4* (CCTCC AB 2015375) is purchased from the China Center for Type Culture Collection (CCTCC, Wuhan, Hubei, China). The recovery of *T4 phage* was performed as follows: *E. coli* BL21 (DE3) was inoculated at a 1:1000 ratio into LB liquid medium and cultured overnight. The following day, *T4 phage* was added to the culture of *E. coli* BL21 (DE3), and the mixture was incubated at 37 °C for 3 days. Then, the infected mixture was centrifuged, filtered, and stored at 4 °C.

### 4.2. DNA Extraction and Sequencing

Genomic DNA of cyanobacteria was extracted using an optimized SDS extraction method [46]. The extracted genomic DNA was used to construct DNA libraries with the SQK-LSK110 kit and the EXP-NBD196 kit (Oxford Nanopore Technologies, Oxford, UK). Libraries that met the quality standards were sequenced on the nanopore PromethION and Illumina NovaSeq 6000 platforms.

Raw nanopore sequencing data were subjected to quality filtering (Q ≥ 7) and length filtering (length ≥ 1600 bp) to obtain valid data for subsequent assembly and analysis. Similarly, raw Illumina sequencing data were processed using fastp for adapter removal, short-read trimming, and low-quality data filtering, yielding high-quality sequencing data. After filtering, the third generation nanopore sequencing generated a total of 982,441,195 bp of clean data for assembly, while the second-generation Illumina sequencing generated 1,276,206,792 bp of clean data. The nanopore data were assembled into high-quality bacterial genome scaffolds (contigs) using Flye, and Illumina data were employed for error correction of the Flye assembly results using Pilon. This hybrid assembly approach effectively mitigated sequence contamination introduced by nanopore’s read-splitting errors.

The assembled genome was subsequently annotated for structural and functional features. Structural annotations included the identification of repetitive sequences, coding genes, non-coding RNAs, genomic islands, and CRISPR regions. Functional annotation was performed using eight major databases: UniProt, KEGG, GO, Pfam, COG, TIGERfams, RefSeq, and NR, providing comprehensive gene function information.

### 4.3. Analysis of the CRISPR System

CRISPR systems were predicted by using the online tool PADLOC [47] with the assembled genome of *M. aeruginosa* FACHB-524. According to the different types of CRISPR-Cas systems, they were named Clusters 1 to 6. Meanwhile, we analyzed the information related to Clusters 1–6 CRISPR-Cas loci with the CRISPRLeader. CRISPRLeader is available at http://www.bioinf.uni-freiburg.de/Software/CRISPRleader/. Protein domains were searched using NCBI’s Conserved Domain Database (CDD).

To explore the evolutionary relationships of the Cas10 (Cmr2) protein in *M. aeruginosa* FACHB-524, homologous protein sequences of Cas10 were retrieved from the NCBI database and used to construct a phylogenetic tree. The tree was generated using the neighbor-joining method based on the Kimura 2-parameter evolutionary model, and node support was evaluated through 1000 bootstrap replicates.

### 4.4. Gene Cloning and Plasmids Construction

Because a manipulable genetic transformation system is lacking for *M. aeruginosa*, *E. coli* was selected for further investigation of the CRISPR systems targeting MaMVs. For this purpose, a type III CRISPR system, the Cluster 4, was selected for construction into prokaryotic expression vectors, which includes eight genes (Csx1, Cas10, Cmr3, Cmr4, Uma2, Cmr5, Cmr1-6, CARF). Additionally, the Cas6 gene, which encodes the crRNA maturation enzyme, was cloned in the same prokaryotic expression vector with the eight Cas genes. The genes were codon optimized and synthesized (Sangon Biotech, Shanghai, China). Then, these genes were amplified using polymerase chain reaction and assembled into the vector pCDFDuet-1 via homologous recombination. The RBS binding sites (5′-3′: ctttaataaggagatatacc or aagtataagaaggagatatac) were placed preceding each gene in the resulting recombinant plasmid. For protein purification, a 10×His tag was added to the C-terminus of Cmr1-6. Each set of four genes is controlled by a T7 promoter to drive the transcription. All final constructs are verified by sequencing, and the recombinant expression vector was named as P-524Cas/Cmr. In addition, a CRISPR array consisting of five 36-nucleotide repeats alternated with three 38-nucleotide spacer sequences and one 40-nucleotide spacer sequence was synthesized and cloned into the pACYC-Duet1 vector, resulting in the P-524S4 plasmid.

According to the construction method described above, the five subunits of the Type III-B effector complex (Cas10, Cmr3, Cmr4, Cmr5, and Cmr1-6) along with Cas6 were cloned into the pCDFDuet-1 vector to form the pCDF-Cmr-α prokaryotic expression vector (Figure S1). The accessory gene Csx1/CARF of the III-B CRISPR system was cloned into the pETDuet vector to obtain the pET-Csx1/CARF expression plasmid. Subsequently, 38 nucleotides (5′–3′: gaattggtttaattgaaattgctacgcaaagtgaagtt) from the *T4 phage gene 34* and the repetitive sequences from Cluster 4 were cloned into the pACYC-Duet1 vector, generating the pACYC T4-34 plasmid (Figure S1). Plasmids pCDFDuet-1, pACYC-Duet1, and pETDuet each harbor distinct replication elements and resistance traits, as detailed in Table S3.

### 4.5. Prokaryotic Expression and Purification of CRISPR-Cas Complexes

The plasmids P-524Cas/Cmr and P-524S4 were sequentially transferred into *E. coli* BL21 (DE3). The positive colonies containing the two plasmids were screened out by PCR detection and cultured in LB liquid medium containing the corresponding antibiotics at 37 °C overnight. On the next day, the bacteria were inoculated into fresh medium at a ratio of 1:100, and incubated with shaking until OD_600_ = 0.6–0.8. Then, IPTG (isopropyl-β-D-thiogalactopyranoside) was added to the medium at a final concentration of 0.3 mM, and induced at 20 °C for 18 h. The bacteria were collected by centrifugation at 5000 g for 10 min, and suspended in binding buffer (50 mM NaH_2_PO_4_, 300 mM NaCl, 10 mM imidazole, pH = 8.0), which were lysed by ultrasonication under the following working conditions: working for 2 s, stopping for 3 s, and a total time of 30 min. After that, the supernatant was collected by centrifugation at 15,000× *g* for 20 min, filtered through a 0.45 μM filter and added to a pre-equilibrated Ni NTA Beads 6FF. The proteins were eluted by gradient increased concentrations (to 0.25 mM) of imidazole. Protein products were analyzed by SDS-PAGE and mass spectrometry analysis.

### 4.6. Mass Spectrometry (MS) Analysis

#### 4.6.1. Sample Preparation

The gel containing protein bands were cut into small pieces of approximately 1 mm^3^ and washed with double-distilled water (ddH₂O) three times. Subsequently, decolorization and dehydration were performed using a solution of 50% acetonitrile (ACN) in 100 mM ammonium bicarbonate (NH₄HCO₃), followed by treatment with 100% ACN. Next, 10 mM dithiothreitol (DTT) in 50 mM NH₄HCO₃ was added, and the mixture was incubated for 1 h at 56 °C with shaking. This was followed by the addition of 55 mM iodoacetamide (IAA) in 50 mM NH₄HCO₃, with shaking for 30 min at 25 °C. Then, 500 µL of ACN was added to rehydrate the pellet, after which trypsin was added, and the reaction was allowed to proceed overnight at 37 °C. At the end of the reaction, 60% ACN and 5% formic acid were added for extraction, and the supernatant was carefully removed. Finally, the peptides were desalted using a ZipTip C18 and then solubilized in 10 μL of loading buffer (0.1% formic acid, 2% ACN).

#### 4.6.2. LC-MS/MS Analysis

Mass spectrometry analysis was performed using a Q Exactive HF-X (Thermo Fisher Scientific, Waltham, MA, USA) liquid chromatography-mass spectrometry (LC-MS) system as described previously [48]. Samples were desalted using a C18 Trap and then separated using a C18 analytical column (75 μm × 250 mm, 2 μm particle size, 100 Å pore size, Acclaim PepMap C18 column, Thermo Fisher Scientific) with gradient elution. The elution time was 65 min, with two mobile phases: A: 0.1% formic acid in water, and B: 80% ACN, 0.1% formic acid. The elution program was as follows: 0% B at 0 min, 4–8% B in 1 min, 8–10% B in 2 min, 10–25% B in 40 min, 25–45% B in 10 min, 45–100% B in 1 min, and 100% B for 11 min. The flow rate of the liquid phase was 300 nL/min.

The mass spectrometry detection results were searched against the coding proteins of *M. aeruginosa*_FACHB-524 and *E. coli* BL21 (DE3).

### 4.7. Phage Infection Assay

The plasmids pCDF Cmr-α, pACYC T4-34, or pET Csx1/CARF were transformed into *E. coli* BL21 (DE3), generating three types of transformants with different combinations: (1) pCDF Cmr-α and pACYC T4-34, (2) pCDF Cmr-α, pACYC T4-34, and pET Csx1, and (3) pCDF Cmr-α, pACYC T4-34, and pET CARF. The transformants containing (1) pCDF Cmr-α and pACYC T4-34 were able to express the Cmr-α-crRNA effector complex from the Cluster 4 CRISPR-Cas system. The transformants containing (2) pCDF Cmr-α, pACYC T4-34, and pET Csx1, as well as (3) pCDF Cmr-α, pACYC T4-34, and pET CARF, were capable of simultaneously expressing both the Cmr-α-crRNA effector complex and the effector proteins Csx1 or CARF (Figure S1).

Next, the three different transformants were inoculated into LB liquid medium containing antibiotics for overnight incubation. The following day, the cultures were diluted 1:100, and cultured until the OD_600_ reached 0.4–0.7, 0.5 mM IPTG was added to induce protein expression. The culture was then incubated at 37 °C for 1 h. Afterward, *T4 phage* was inoculated, and the culture continued to incubate at 37 °C. Six hours post-infection, the *E. coli* culture and *T4 phage* mixture were collected and centrifuged. The supernatant was then serially diluted in a 10-fold gradient using SM buffer (100 mM NaCl, 10 mM MgSO_4_, and 50 mM Tris-HCl, pH = 7.5). Five μL of the diluted *T4 phage* solution was spotted onto a bilayer plate containing *E. coli* BL21 (DE3) to monitor plaque formation. The *E. coli* BL21 (DE3) bilayer plates were prepared as follows: *E. coli* BL21 (DE3) was cultured overnight, then diluted 1:100 in LB medium. The culture was grown to an OD_600_ of 0.6–0.8. A 500 μL aliquot of the culture was mixed with 10 mL of LB medium containing 0.75% agar and overlaid onto a pre-poured LB agar plate containing 1.5% agar.

## 5. Conclusions

The composition and function of the CRISPR systems of *M. aeruginosa* FACHB-524 were investigated by genome re-sequencing, bioinformatical analysis, and functional validation. Two type I and three type III-B CRISPR systems were depicted and two of them showed the ability to target MaMVs, indicating a complex interaction between *M. aeruginosa* and its cyanophages. By using the model bacteria *E. coli*, a type III-B system was reconstructed and showed anti-phage activity. The results significantly enhanced our understanding of the CRISPR systems in *Microcystis*, providing a valuable platform for further investigation of their underlying mechanisms. The platform also aids in uncovering the details of algal immune responses, which may contribute to the development and application of artificial cyanophages for controlling cyanobacterial blooms.

## Figures and Tables

**Table 1:** General characteristics of *M. aeruginosa* FACHB-524 genome.

Features	*M. aeruginosa* FACHB-524-Genome	*M. aeruginosa* FACHB-524 Unclassified-1	*M. aeruginosa* FACHB-524 Unclassified-2	*M. aeruginosa* FACHB-524 Unclassified-3	*M. aeruginosa* FACHB-524 Unclassified-4
Type	Chromosome	Plasmid	Plasmid	Plasmid	Plasmid
Genome size	4,933,804 bp	109,955 bp	14,853 bp	8672 bp	7153 bp
GC content	42.49%	42.96%	40.85%	43.20%	44.65%
CDS numbers	4828	138	19	10	8
rRNA operon	4	0	0	0	0
tRNA genes	44	0	0	0	0
tmRNA	1	0	0	0	0
misc_rna	19	0	0	0	0

**Table 2:** Overview of the CRISPR-Cas loci presented in the genome of *M. aeruginosa* FACHB-524.

Cas Operon Type	No. of CRISPR Arrays	Genome Name	Position in the Genome	Repeat Consensus	No. of Spacers	Strand
Type I-B (Cluster 1)	1	*M. aeruginosa* FACHB-524 unclassified-1	38,665–51,953	GTGTACTAACCTT TGATGCGTAAGGC GTTGATCAC	34	Reverse
Type I-D (Cluster 2)	4	*M. aeruginosa* FACHB-524-genome	2,962,948–3,010,089	GTTCCAATTAATC TTAAACCCTACTA GGGATTGAAAC	200	Forward
Type III-B (Cluster 3)	2	*M. aeruginosa* FACHB-524-genome	905,120–915,905	CTTGCTTCCAATT CGTGAAGCGTATG AATGGAAAC	25	Forward
Type III-B (Cluster 4)	1	*M. aeruginosa* FACHB-524-genome	2,333,504–2,346,193	CTCTCTACTCGCT AGAGAAATTAATT GAATGGAAAC	21	Reverse
Type III-B (Cluster 5)	2	*M. aeruginosa* FACHB-524-genome	2,463,006–2,470,242	CCTTACCTATTAG GTCAAATAGGATT AGTTGGAA	4	Reverse

## Data Availability

Data are contained within the article and Supplementary Materials. And the genome sequences of the *Microcystis aeruginosa* FACHB-524 have been submitted into NCBI BioSample database under accession number SAMN46003490.

## Supplementary Materials

The supporting information can be downloaded at: https://www.mdpi.com/article/10.3390/ijms26041554/s1.

## Abbreviation

*M. aeruginosa*	*Microcystis aeruginosa*
CRISPR	Clustered regularly interspaced short palindromic repeat
Cas	CRISPR-associated proteins
Cmr	Cas module RAMP (Receptor activity-modifying proteins)
crRNA	CRISPR RNA
MaMV	*M. aeruginosa* myovirus
*T4*	*Enterobacteria phage T4*
