# Supplementary material for: Characterization of Five CRISPR Systems in Microcystis aeruginosa FACHB-524 with Focus on the In Vitro Antiviral Activity of One CRISPR System
Source: Int J Mol Sci. 2025 Feb 12;26(4):1554. doi: 10.3390/ijms26041554 (PMC11855584; doi:10.3390/ijms26041554)
Supplement: Supplementary file 1 [file ijms-26-01554-s001.zip › ijms-3438308-supplementary.pdf]

International Journal of *Molecular Sciences*

MDPI

Article

Characterization of Five CRISPR Systems in *Microcystis aeruginosa* FACHB-524 with Focus on the In Vitro Antiviral Activity of One CRISPR System

Mengjing Zeng [1,2], Qi-Ya Zhang [2] and Fei Ke [2,*]

[1] College of Advanced Agricultural Sciences, University of Chinese Academy of Sciences, Beijing 100049, China; zengmengjing@ihb.ac.cn
[2] Institute of Hydrobiology, Chinese Academy of Sciences, Wuhan 430072, China; zhangqy@ihb.ac.cn
* Correspondence: kefei@ihb.ac.cn; Tel.: +86-027-68780002

Supplemental Data

Supplemental Figures

Figure S1 (related to Figure 5)**:** Different transformants (pCDF Cmr-α-pACY T4-34, pCDF Cmr-α-pET-Csx1, pCDF Cmr-α-pET-CARF, pCDF Cmr-α-pACYC T4-34-pET-Csx1, and pCDF Cmr-α-pACYC T4-34-pET-CARF) were used to express various strategies of the III-B type CRISPR system.

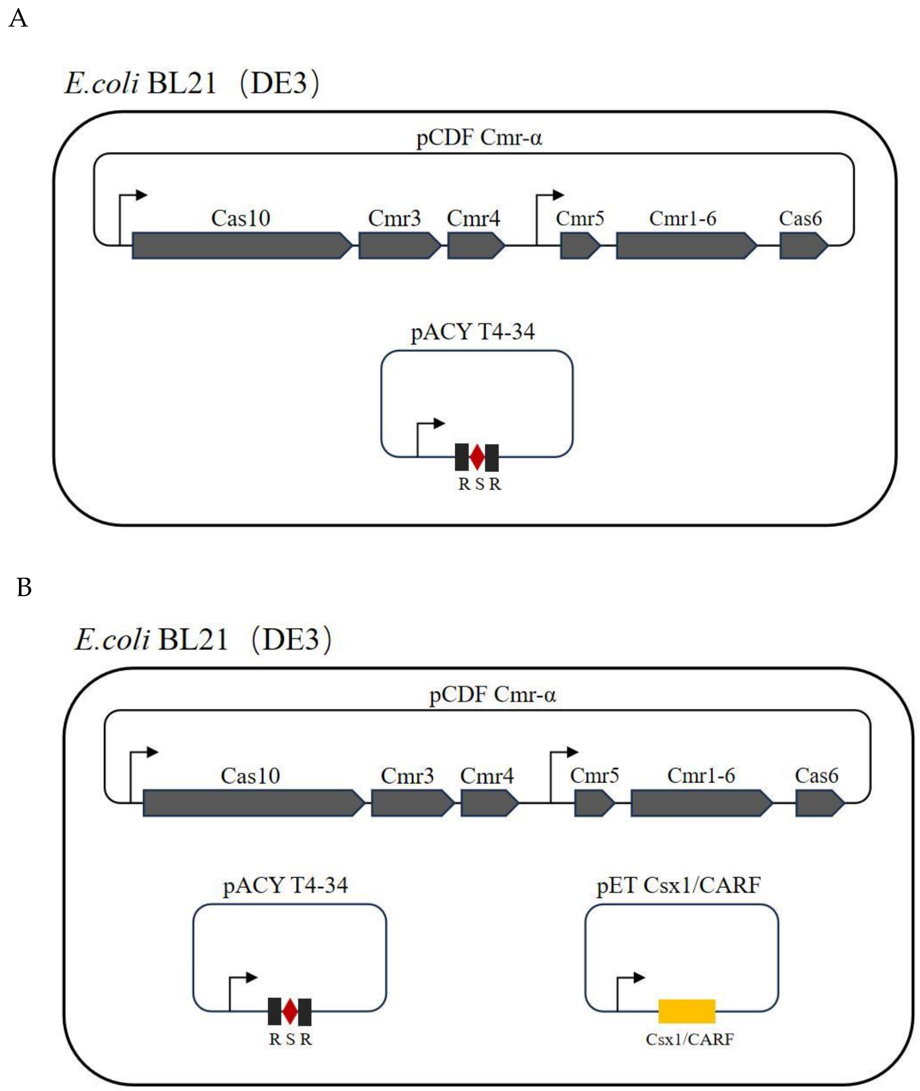

Figure S1. A. pCDF Cmr-*α*-pACY T4-34. Transformants containing both plasmids were able to express the Cmr-*α* effector complex. B. pCDF Cmr-*α*-pACYC T4-34-pET Csx1/CARF. Transformants containing these three plasmids were able to express the Cmr-*α* effector complex and the accessory protein Csx1/CARF. The black arrows indicate the T7 promoter. pCDF Cmr-*α*, pACY T4-34, and pET Csx1/CARF represent the names of the constructed expression vectors. R denotes the repeat sequences of the CRISPR system, derived from the III-B type (Cluster 4) of *Microcystis aeruginosa* FACHB-524, and S derived from the *T4 phage* gene *34*.

Supplemental Tables

Table S1 (related to Figure 2)**:** The amino acid sequences of Cas10/Cmr3/Cmr4/Cmr5/Cmr6 homologues of Clusters 3, 4 and 5 were aligned by BLASTP.

Comparison	identitiy
Cluster 3-Cas10 vs Cluster 4-Cas10	27.63 %

Cluster 3-Cas10 vs Cluster 5-Cas10	40.71 %
Cluster 4-Cas10 vs Cluster 5-Cas10	27.04 %
Cluster 3-Cmr3 vs Cluster 4-Cmr3	44.93 %
Cluster 3-Cmr3 vs Cluster 5-Cmr3	28.42 %
Cluster 4-Cmr3 vs Cluster 5-Cmr3	22.83 %
Cluster 3-Cmr4 vs Cluster 4-Cmr4	28.32 %
Cluster 3-Cmr4 vs Cluster 5-Cmr4	50.34 %
Cluster 4-Cmr4 vs Cluster 5-Cmr4	30.71 %
Cluster 3-Cmr5 vs Cluster 4-Cmr5	0 %
Cluster 3-Cmr5 vs Cluster 5-Cmr5	51.75 %
Cluster 4-Cmr5 vs Cluster 5-Cmr5	0 %
Cluster 3-Cmr1-6 vs Cluster 4-Cmr1-6	22.43 %
Cluster 3-Cmr1-6 vs Cluster 5-Cmr1-6	40.69 %
Cluster 4-Cmr1-6 vs Cluster 5-Cmr1-6	21.56 %
Cluster 3-Cas10 vs Cluster 4-Cas10	27.63 %

Table S2 (related to Figure 4)**:**

The mass spectrometry results of Cas10, Cmr1-6, Cmr3, Cmr4, and Cmr5:

Accession	Description	Sum PEP Score	Coverage [%]	# Peptides	# PSMs	# Unique Peptides	# AAs	MW [kDa]	calc. pI	Score Sequest HT: Sequest HT	# Peptides (by Search Engine): Sequest HT	# Protein Groups
WP_123230762.1	type III-B CRISPR-associated protein Cas10/Cmr2 [Microcystis aeruginosa]	761.143	81	101	621	101	1003	115.2	6.9	1789.15	101	1
WP_123230764.1	type III-B CRISPR module RAMP protein Cmr6 [Microcystis aeruginosa]	486.698	73	52	343	52	667	75.4	9.41	692.38	52	1
WP_123230763.1	CRISPR-associated protein Cmr3 [Microcystis aeruginosa]	149.659	61	26	107	26	374	42.8	8.25	293.32	26	1
WP_002737749.1	type III-B CRISPR module RAMP protein Cmr4 [Microcystis aeruginosa]	264.655	78	27	208	27	259	29.2	7.11	561.86	27	1
WP_002735800.1	hypothetical protein(Cmr5) [Microcystis aeruginosa]	114.325	61	14	66	14	129	14.6	5.31	204.39	14	1

The mass spectrometry results of Cas10, Cmr1-6, Cmr3, Cmr4, and Cmr5 were obtained by searching and comparing the *Microcystis_aeruginosa*_FACHB524_*Microcystis*_phage_MaMVDC.fasta and *Escherichia coli* (strain B BL21-DE3). fasta databases.

Table S3: The origin of replication (ori) and resistance gene of the plasmids used in this study and the resistance genes they carry.

Plasmid	Origin of replication	resistance gene
pCDFDuet-1	CloDF13	streptomycin
pACYC-Duet1	p15A	Chloramphenicol
pETDuet	ColE1	Ampicillin
